# Weaning Induces Stress-Dependent DNA Methylation and Transcriptional Changes in Piglet PBMCs

**DOI:** 10.3389/fgene.2021.633564

**Published:** 2021-02-05

**Authors:** Ryan J. Corbett, Andrea M. Luttman, Kaitlin E. Wurtz, Janice M. Siegford, Nancy E. Raney, Laura M. Ford, Catherine W. Ernst

**Affiliations:** ^1^Genetics and Genome Sciences Graduate Program, Michigan State University, East Lansing, MI, United States; ^2^Department of Animal Science, Michigan State University, East Lansing, MI, United States

**Keywords:** epigenetics, DNA methylation, pig, weaning, gene expression, *NR3C1*

## Abstract

Changes to the epigenome, including those to DNA methylation, have been proposed as mechanisms by which stress can induce long-term physiological changes in livestock species. Pig weaning is associated with dietary and social stress, both of which elicit an immune response and changes to the hypothalamic–pituitary–adrenal (HPA) axis. While differential methylation following stress has been assessed in model organisms, it remains poorly understood how the pig methylome is altered by stressors in production settings. We quantified changes in CpG methylation and transcript abundance in piglet peripheral blood mononuclear cells (PBMCs) following weaning and also assessed differential patterns in pigs exhibiting high and low stress response as measured by cortisol concentration and lesion scores. Blood was collected from nine gilt piglets 24 h before and after weaning, and whole-genome bisulfite sequencing (WGBS) and RNA-sequencing were performed on six and nine animals, respectively, at both time points. We identified 2,674 differentially methylated regions (DMRs) that were enriched within promoters of genes associated with lymphocyte stimulation and transcriptional regulation. Stress groups displayed unique differential methylation and expression patterns associated with activation and suppression of T cell immunity in low and high stress animals, respectively. Differential methylation was strongly associated with differential expression; specifically, upregulated genes were enriched among hypomethylated genes. We observed post-weaning hypermethylation of the glucocorticoid receptor (*NR3C1*) promoter and a significant decrease in *NR3C1* expression (*n* = 9, *p* = 6.1 × 10^–3^). Our results indicate that weaning-associated stress elicits genome-wide methylation changes associated with differential gene expression, reduced T cell activation, and an altered HPA axis response.

## Introduction

Livestock animals experience numerous stressors throughout their lifetime that result in short- and long-term effects on physiology and performance (Yahav and Hurwitz, 1996; Geverink et al., 1998; Molenaar et al., 2011; Li et al., 2017; Johnson et al., 2018). Weaning represents a period of dietary and social stress in pig production systems with acute effects on digestive physiology and immune response (Lallès et al., 2004). In addition to direct effects of weaning, piglets are exposed to unfamiliar individuals in nursery pens, which results in aggressive encounters and skin lesion development (Wurtz et al., 2017). Overall, blood lymphocyte concentrations have been shown to be significantly reduced in weaned pigs (Kick et al., 2012), and additional psychosocial stress experienced during weaning has been associated with lower concentrations of T cell subpopulations (Tuchscherer et al., 2009). One reliable indicator of stress response following pig weaning and mixing is lesion counts, as this measure has been shown to be associated with aggressive behavior, hypothalamic–pituitary–adrenal axis (HPA) activity, and risk of infection, as well as negatively associated with immunocompetence (Fernandez et al., 1994; Morrow-Tesch et al., 1994; Turner et al., 2006, 2009). Weaning stress can have long-term consequences on gut health and disease susceptibility (reviewed in Moeser et al., 2017), yet the molecular mechanisms underlying these relationships remain poorly characterized. It has been proposed that epigenetic mechanisms may link stress from environmental stimuli to lasting changes in gene expression and physiology (Tzschentke and Basta, 2002; González-Recio et al., 2015).

DNA methylation is an epigenetic modification involving the enzymatic addition of a methyl group to the 5-carbon of cytosine rings, producing 5-methylcytosine. Methylation occurs almost exclusively at CpG dinucleotides in mammals and has context-specific associations with gene expression. In gene promoters and intronic enhancers, methylation generally functions to decrease levels of transcription through the alteration of transcription factor binding sites or the recruitment of transcriptional repressors (Bird and Wolffe, 1999; Varley et al., 2013; Greenberg and Bourc’his, 2019). Numerous studies have assessed DNA methylation patterns in peripheral blood mononuclear cells (PBMCs), as these can be repeatedly obtained from the same subjects and provide indications of alteration in stress response pathways; for instance, human studies have found increased levels of stress exposure to be associated with increased glucocorticoid receptor (*NR3C1*) methylation and decreased *NR3C1* expression in blood (Oberlander et al., 2008; Perroud et al., 2011; González Ramírez et al., 2020). DNA methylation studies have been performed in pig tissues in response to different stressors (Hao et al., 2016; Wang et al., 2017). However, assessment of DNA methylation in the pig in response to natural production stressors has not been extensively studied nor has the association between differential methylation and gene expression.

In the current study, we sought to identify differentially methylated regions (DMRs) and differentially expressed genes (DEGs) associated with weaning. We also assessed differential methylation and expression separately in pigs exhibiting high and low levels of weaning stress as determined by changes in serum cortisol concentration and post-weaning lesion counts. Lastly, we looked specifically at the effect of weaning on *NR3C1* methylation and expression as an indicator of alterations in the HPA axis response.

## Materials and Methods

### Sample Collection

Blood was sampled from nine crossbred gilts (four and five from two litters) weaned at 28–29 days of age. At weaning, littermates were separated into nursery pens containing six gilts of various litters. Samples were collected 24 h before and 24 h after weaning, and body lesions were counted immediately before and 24 h after weaning by the same trained counter (Wurtz et al., 2017). Blood was collected from each animal per time point in Vacutainer whole blood collection tubes with no additive (Becton Dickinson) for serum isolation and Vacutainer tubes containing freeze-dried sodium heparin (VWR) for PBMC isolation. PBMCs were isolated from each animal at both time points following centrifugation in ACCUSPIN^TM^ System-Histopaque-1077^®^ tubes (Sigma-Aldrich) according to manufacturer’s instructions. Serum cortisol concentrations were measured using the DetectX Cortisol Enzyme Immunoassay from Serum and Plasma Kit (Arbor Assays). Optical density was measured at 450 nm using a microplate reader, and readings were converted into ng/ml concentrations using Arbor Assays software. We assessed changes in serum cortisol concentration and post-weaning lesion counts as phenotypic measurements of stress response. High stress (HS) and low stress (LS) animals were visually selected as those at the extremes of the two-dimensional plot of the two phenotypes and further subjected to DNA methylation analyses (Supplementary Figure 1).

### Nucleic Acid Isolation and Sequencing

DNA was isolated from six pre- and six post-weaning PBMC samples using the PureLink Genomic DNA Kit. Prior to library preparation, DNA samples were spiked with unmethylated lambda phage DNA (5 ng lambda DNA/1 μg sample DNA) to assess sodium bisulfite conversion. DNA was sodium bisulfite converted using the Zymo EZ DNA Methylation-Gold Kit. Eight of the 12 libraries were prepared using the Kapa Hyper Prep DNA Kit (Roche), and the remaining four libraries were prepared using the Swift Biosciences Accel-NGS Methyl-Seq Library Kit. WGBS was performed on an Illumina HiSeq 4000 instrument in 2 × 150 PE format at an average depth of 18X per sample.

Peripheral blood mononuclear cell RNA from all animals at both time points was isolated using the miRNeasy Mini Kit (Qiagen). For eight of the 18 RNA samples, ribosomal RNA (rRNA) was depleted using the Illumina Ribo-Zero Gold Kit; the remaining RNA samples were subject to rRNA and globin RNA depletion using Illumina Ribo-Zero Plus Kit. All sequencing libraries were prepared using the Illumina TruSeq Total RNA Library Preparation Kit. RNA-sequencing (RNA-seq) was performed on an Illumina HiSeq 4000 instrument in 2 × 150 PE format.

### WGBS Bioinformatics

Whole-genome bisulfite sequencing reads were trimmed using Trimmomatic (Bolger et al., 2014) to remove low-quality bases and reads (parameters: LEADING:25 TRAILING:25 AVGQUAL:20 MINLEN:30). Libraries prepared using the Roche kit were subject to the removal of the first six bases from forward and reverse reads (HEADCROP:6), while libraries prepared using the Swift Biosciences kit were subject to removal of the first 10 bases from forward reads and first 15 bases from reverse reads (HEADCROP:10/15) per the manufacturer’s instructions.

Trimmed reads were aligned to the *Sus scrofa* reference genome (v11.1) using Bismark v0.18.1 (Krueger and Andrews, 2011). The genome_preparation command with default parameters was used to create a bowtie2 index of an *in silico* bisulfite-converted reference genome, after which the alignment was performed using the Bismark command (parameters: –maxins 1000 –score_min L,0,-0.6 –unmapped). Reads that were unpaired following trimming were merged with unmapped forward reads and remapped as single-end reads using the same parameters. We assessed sodium bisulfite conversion efficiency of samples by mapping reads to a lambda genome bowtie2 index with default parameters. Among reads aligning to the lambda genome, the percentage of methylated cytosines was subtracted from 100 to calculate bisulfite conversion efficiency. Due to sub-optimal conversion in some samples, we ran Bismark’s filter_non_conversion command with default parameters to remove reads that contained >3 consecutive methylated non-CpG cytosines. Conversion rates were >99.5% in all libraries following this filtering. Remaining reads were deduplicated using Bismark’s deduplicate_bismark command with default parameters, and the methylation_extractor command was used to calculate methylation rates at all CpGs in the *S. scrofa* genome using default parameters.

Differential methylation analysis between stages was performed using the *methylKit* R package v.1.6.3 (Akalin et al., 2012). For each sample, we discarded CpGs in the 99.5th percentile of coverage to exclude potential PCR duplicates. CpGs within 1 kb non-overlapping windows were consolidated, and average CpG methylation rates were calculated. Only regions with coverage of at least 20 reads in >4 samples per treatment were retained for further analysis. Logistic regression models were fitted for each region accounting for the fixed effects of library prep and animal within library prep, and random effect of litter to test if stage (post- versus pre-weaning) had a significant effect on the log odds ratio of regional CpG methylation rate. DMRs were classified as those with methylation difference >10% and FDR < 0.05. Separate post-weaning differential methylation analyses were also run for LS and HS animals using the same methods.

Differentially methylated regions were annotated with respect to their overlap with gene features to identify differentially methylated genes (DMGs). Genes were classified as promoter- or gene body-DMGs if they contained a DMR in their promoter [2 kb up- or downstream of transcription start site (TSS)] or gene body, respectively. DMRs not overlapping a gene were classified as intergenic. DMG lists were submitted for gene set enrichment analysis (GSEA) using GOrilla software (Eden et al., 2009).

### RNA-Sequencing Bioinformatics

RNA-sequencing reads were trimmed of adapters and low-quality bases using Trimmomatic (HEADCROP:10 LEADING:25 TRAILING:25 AVGQUAL:20 MINLEN:30).

Trimmed reads were aligned to the *S. scrofa* reference genome using TopHat2 (Kim et al., 2013) (parameter: –library-type “fr-firststrand”), and gene counts were obtained using HTSeq-count (Anders et al., 2015) (parameters: -m intersection-non-empty -i gene_id -t exon -s reverse).

Differential expression analyses were performed using the *DESeq2* R package (Love et al., 2011). A negative binomial model was fitted that tested for the effect of stage and corrected for the effects of litter, library preparation, and animal within library preparation. DEGs were classified as those with FDR < 0.05, regardless of log2-fold change. DEGs were submitted for GSEA using GOrilla to identify enriched GO terms. Four separate *DEseq2* analyses were performed for: (1) all animals (*n* = 9/stage), (2) HS animals and (3) LS animals (*n* = 3/stage), and (4) animals with WGBS data (*n* = 6/stage).

### Assessment of NR3C1 Methylation and Expression

We assessed *NR3C1* promoters for regional and site-specific methylation differences, and PBMC *NR3C1* transcript abundance was measured by RNA-seq and RT-qPCR. Total RNA was reverse transcribed using the High Capacity cDNA Reverse Transcription Kit (Applied Biosystems). *B2M* and *GAPDH* were used as reference genes due to their reported stable expression in PBMCs (Cinar et al., 2013; Wang et al., 2014). Assays were performed in triplicate on a StepOnePlus Real-time PCR Instrument (Applied Biosystems) using 5 μl cDNA (50 ng total), 1 μl TaqMan Gene Expression Assay (Applied Biosystems Assay Nos. Ss03378868_u1, Ss03374854_g1, and Ss03391154_m1 for *NR3C1*, *B2M*, and *GAPDH*, respectively), 10 μl TaqMan Fast Advanced Mastermix (Applied Biosystems), and 4 μl water. Reaction conditions were 50°C for 2 min and 95°C for 2 min, followed by 40 cycles of 95°C for 1 s and 60°C for 20 s. Delta Cts (dCts) were obtained for each sample by subtracting the geometric mean of the reference gene Cts from the *NR3C1* Ct, and a paired *t*-test was performed to assess the significance of stage on dCts. Fold change in abundance at post- versus pre-weaning was calculated using the 2^–ΔΔCt^ method.

## Results

### Serum Cortisol and Lesion Count Measurements Identify Low- and High-Stress Animals Following Weaning

We measured serum cortisol concentrations and skin lesion counts before and after weaning in nine gilts in order to assess differential stress response. Percent change in serum cortisol concentration was significantly positively correlated with post-weaning lesion counts (*r* = 0.70, *p* = 0.036; Supplementary Figure 1). We designated the three animals exhibiting the lowest and highest values of these parameters as LS (two gilts from litter 98 and one from litter 102) and HS (one gilt from litter 98 and two from litter 102), respectively.

### Weaning Differential Methylation Is Associated With Lymphocyte Immune Response Genes

**FIGURE 1:**
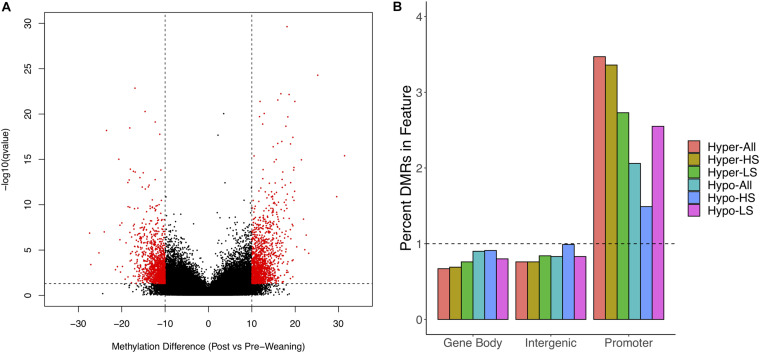
Differential methylation in post- versus pre-weaning PBMCs. **(A)** Volcano plot of methylation difference against –log10 (*q*-value). Red dots indicate significant differentially methylated regions (DMRs). **(B)** Enrichment of hypomethylated and hypermethylated DMRs for All, HS, and LS pigs in gene features. Horizontal line indicates expected relative proportion of DMRs in feature if no enrichment (i.e., 1). HS, high stress; LS, low stress.

We obtained 128–194M WGBS reads across samples, of which 86–89% uniquely aligned to the *S. scrofa* reference genome (Supplementary Table 1). We achieved sufficient coverage to assess differential methylation post- versus pre-weaning at 972,067 1 kb regions. We observed clustering of samples based on litter (data not shown) and thus corrected for this as a random effect in our differential methylation analysis. We identified 2,674 DMRs between stages when considering all animals, of which 1,363 DMRs were hypermethylated and 1,311 DMRs were hypomethylated post-weaning (Figure 1A and Supplementary Tables 2,3). We annotated DMRs along with all tested regions to gene features and observed a 3.4-fold and 2.1-fold overrepresentation of hyper- and hypo-DMRs in gene promoters, respectively (Figure 1B). Conversely, there was a depletion of DMRs in gene bodies and intergenic regions.

We also assessed post- versus pre-weaning differential methylation separately in LS and HS animals and identified unique patterns between groups. HS animals possessed a greater number of DMRs overall (9,945 vs. 6,141); however, while HS animals had roughly the same number of hyper- and hypo-DMRs (4,938 and 5,007, respectively), LS animals had a greater proportion of hypo-DMRs (*n* = 3,473) relative to hyper-DMRs (*n* = 2,668). Similar to DMRs among all animals, HS hyper-DMRs were more overrepresented in promoters than were HS hypo-DMRs (Figure 1A), while LS hyper- and hypo-DMRs were overrepresented in promoters at similar magnitudes. These results indicate that differential methylation at weaning is strongly associated with gene promoters and suggest that the proportions of hyper- and hypo-DMRs are dependent on stress level.

To determine if differential methylation influences genes involved in similar biological processes, we submitted DMGs for enrichment analysis (Table 1). Among all animals, promoter-DMGs were enriched for T cell- and plasma cell-specific processes. Genes hypermethylated in their promoters included interleukin 2 receptor alpha (*IL2RA*) and LIM domain only 1 (*LMO1*), whereas hypomethylated genes included several involved in T cell proliferation (*CD3E* and *TNFSF14*) and apoptosis (*LGALS1*). While hypermethylated gene body-DMGs were not enriched for specific processes related to immunity, we identified several such processes enriched among hypomethylated DMGs including “Positive regulation of lymphocyte differentiation” and “B cell receptor signaling pathway.”

**TABLE 1:** Enriched GO terms among promoter- and gene body-DMGs.

GO term	Enrichment	*P*-value	Genes
**Hypermethylated promoter-DMGs**			
Catecholamine secretion	34.05	8.60E-04	*MECP2, NISCH*
Regulation of T cell homeostatic proliferation	34.05	8.60E-04	*IL2RA, LMO1*
**Hypomethylated promoter-DMGs**			
T cell costimulation	7.44	5.30E-04	*PTPN6, TNFSF14, LGALS1, CD3E, PIK3R1*
Lymphocyte costimulation	7.28	5.8E-04	
Plasma cell differentiation	43.66	6.98E-04	*LGALS1, ITM2A*
**Hypomethylated gene body-DMGs**			
Positive regulation of lymphocyte differentiation	3.62	2.07E-04	*PIK3R6, RASGRP1, IL4R, TOX, ZBTB7B, IL2RA, IL12RB1, PTPRC, INPP5D, ZMIZ1, RUNX1*
Regulation of response to stimulus	1.28	2.68E-04	157 genes
Positive regulation of leukocyte differentiation	2.92	3.06E-04	*PIK3R6, IL4R, TOX, IL2RA, TRIB1, IL12RB1, GNAS, INPP5D, RUNX1, RASGRP1, ZBTB46, ZBTB7B, PTPRC, ZMIZ1*
Positive regulation of hemopoiesis	2.68	3.11E-04	*PIK3R6, IL4R, TOX, IL2RA, TRIB1, IL12RB1, GNAS, INPP5D, RUNX1, RASGRP1, ZBTB46, ZBTB7B, PTPRC, ZMIZ1, STAT3, ETS1*
Immune response-activating cells surface receptor signaling pathway	2.31	4.34E-04	*VAV3, ELMO2, ICAM2, MAPK1, BLK, INPP5D, PSMB7, MYO1G, CYFIP2, ACTB, LCP2, PRKCB, BTRC, EIF2B2, MYO1C, NFATC2, PTPRC, ARPC1B, CD247, PDE4B*
B cell receptor signaling pathway	5.50	6.17E-04	*VAV3, ELMO2, ICAM2, MAPK1, BLK, PTPRC, NFATC2*

We also identified shared and unique enriched GO terms among DMGs in LS and HS animals (Supplementary Table 4). In both groups, post-weaning promoter hypomethylation was enriched in genes involved in leukocyte differentiation and activation (Supplementary Tables 4A,E). Post-weaning hypomethylated genes in the LS group were also enriched for T cell-related processes (“alpha-beta T cell receptor complex” and “regulation of gamma-delta T cell activation”). Conversely, terms related to T cells (“alpha-beta T cell activation” and “T cell differentiation”) were enriched among hypermethylated genes in the HS group (Supplementary Table 4F). As gene hypomethylation is generally associated with gene activation, these data suggest that weaning stress level is negatively associated with T cell activity in part through differences in DNA methylation. To further test this hypothesis, we assessed T cell co-receptor genes for post-weaning differential methylation in both stress groups. We identified opposing differential methylation in these genes between stress groups (Table 2). Regions in *CD3E*, *CD3D*, *CD3G*, and *CD4* were hypomethylated post-weaning in the LS group but not DM in the HS group, while a *CD8B* region was hypermethylated post-weaning in the HS group and not DM in the LS group.

**TABLE 2:** Weaning differential methylation and expression of T cell co-receptor genes.

Gene	DMR location	Gene feature	Methylation difference (post vs. pre)			Log2 fold change (post vs. pre-weaning)		
			All	LS	HS	All	LS	HS
*CD3E*	9:45620001-45621000	Promoter	−9.13	−13.17	−6.02	0.076	0.064	0.024
	9:45621001-45622000	Promoter	−16.43*	**−19.64**	**−17.50**			
	9:45624001-45625000	Gene body	−6.63	**−13.63**	2.98			
*CD3D*	9:45645001-45646000	Promoter	−5.08	**−18.71**	2.95	**0.273**	0.165	0.081
*CD3G*						−0.460	−0.528	−0.588
*CD4*	5:63916001-63917000	Promoter	−5.39	**−17.70**	6.06	−0.122	−0.143	−0.387
*CD8A*	–	–	–	–	–	**−0.311**	−0.079	−0.59
*CD8B*	3:57970001-57971000	Promoter	**16.50**	**15.17**	**17.49**	**−0.80**	−0.251	**−1.67**
	3:57988001-57989000	Gene body	11.93	−0.81	**20.57**			

*Boldface indicates significant differential methylation/expression.*

### Weaning Differential Gene Expression Is Associated With Differential Gene Methylation

We assessed PBMC gene expression in nine gilts via RNA-seq and obtained 62–108M reads per sample (Supplementary Table 5). We identified 13,580 expressed genes and 1,480 DEGs between stages (Supplementary Table 6; 721 upregulated and 759 downregulated post-weaning). Numerous GO terms were enriched among upregulated genes, the most significant of which were related to immune, inflammatory, and stress response (Supplementary Table 7A). Downregulated genes were enriched for terms related to transcriptional and post-transcriptional regulation (Supplementary Table 7B).

Differential expression analysis was also performed on LS and HS animals separately. A larger number of weaning DEGs were identified in the LS group (134 upregulated and 42 downregulated post-weaning) compared to the HS group (49 genes upregulated and 13 downregulated). DEGs were submitted for GSEA, and only upregulated genes contained enriched GO terms. There were numerous terms enriched among both sets of upregulated genes (e.g., “Inflammatory response” and “Positive regulation of cytokine production”), but unique GO terms were also enriched (Supplementary Tables 7C,D). LS upregulated genes were enriched for terms related to viral response, type I interferon signaling, and NIK/NF-κB signaling. HS upregulated genes were enriched for terms related to apoptosis and negative regulation of CD4-positive, alpha-beta T cell proliferation and activation.

To determine the degree of overlap between DEGs and DMGs, we identified DEGs between stages in the same six animals for which methylation data were generated. There was a total of 387 DEGs (275 upregulated and 112 downregulated), and these were significantly enriched among DMGs (Supplementary Figure 2). Twenty-eight DEGs were also promoter-DMGs, and there was particular enrichment for upregulated genes among hypomethylated promoter-DMGs (*p* = 3.56 × 10^–3^; Supplementary Figure 2A). Additionally, 29 DEGs were also gene body-DM, and there was again enrichment for upregulated genes among hypomethylated gene body-DMGs (*p* = 0.021; Supplementary Figure 2B). There is thus evidence that differential methylation is strongly associated with differential expression in post-weaning piglet PBMCs, and specifically that hypomethylation is associated with increased gene expression.

Because T cell co-receptor genes exhibited unique differential methylation patterns between stress groups, we determined the extent to which such patterns associated with differential expression between HS and LS animals. *CD8B*, which was gene body-hypermethylated only in the HS group post-weaning, was also significantly downregulated in the HS group (Table 2; log2FC = −1.67) but not in the LS group (log2FC = −0.25). *CD4* exhibited post-weaning promoter hypomethylation in the LS group but not in the HS group and exhibited more decreased expression in the HS group (log2FC = −0.387) although this difference was not statistically significant. Changes in the expression of genes with associated differences in regional methylation suggest that such regions may harbor regulatory elements that dictate stress-dependent T cell gene expression.

### *NR3C1* Differential Methylation and Expression Is Indicative of Altered HPA Axis Response

**FIGURE 2:**
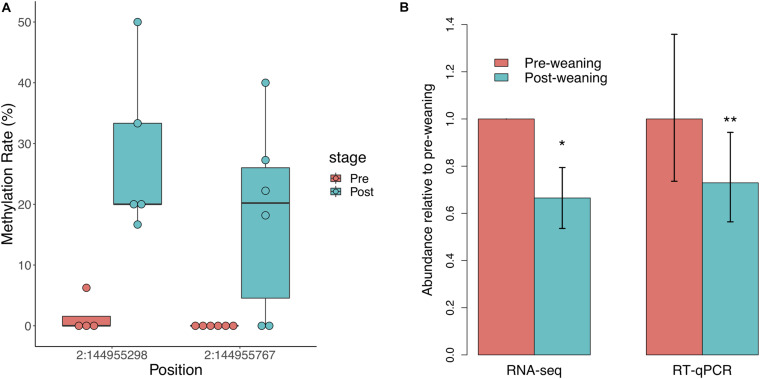
Glucocorticoid receptor (*NR3C1*) gene methylation and expression in response to weaning. **(A)** Two CpG sites in the *NR3C1* promoter (797 and 328 bp downstream of TSS) are significantly hypermethylated post-weaning (*n* = 6 animals/stage). **(B)** NR3C1 transcript abundance is significantly reduced post-weaning, as measured by RNA-sequencing and RT-qPCR (*n* = 9 animals/stage). *= < 0.05, **= < 0.01.

We did not observe significant regional differences in CpG methylation associated with weaning in either of the two *NR3C1* promoters in the pig genome. We thus assessed individual CpG methylation differences and identified two differentially methylated CpGs (DMCs), both of which were hypermethylated post-weaning (Figure 2A). These DMCs lie 797 and 328 bp downstream of the first *NR3C1* TSS and exhibited 17 and 21% increases in methylation post-weaning, respectively. We observed a corresponding decrease in *NR3C1* transcript abundance via RNA-seq (Figure 2B; log2FC = −0.588, *p* = 0.026), which was also validated via RT-qPCR (log2FC = −0.455, *p* = 6.1 × 10^–3^). These results recapitulate findings in human and mouse studies that stress exposure is associated with *NR3C1* promoter hypermethylation and decreased expression in peripheral tissues.

## Discussion

This study has identified epigenetic alterations in pigs as a response to weaning stress by assessing DNA methylation patterns in piglet PBMCs prior to and after weaning and mixing with unfamiliar individuals. By selecting animals displaying extremes in post-weaning serum cortisol change and skin lesion counts, we were able to assess not only the overall effect of weaning on DNA methylation in PBMCs but also how responses varied depending on stress level. PBMCs are a valuable peripheral cell type to study in the context of weaning for numerous reasons; first, they consist primarily of monocytes and lymphocytes whose activity, proliferation, and differentiation has been shown to be significantly altered by weaning-associated stress (Blecha et al., 1983; Niekamp et al., 2007; Kick et al., 2012). Furthermore, PBMC expression and methylation of genes involved in the HPA axis—namely *NR3C1*—have been shown to be suitable “surrogates” for measurement of gene activity in neuronal tissues (González Ramírez et al., 2020).

We observed global CpG methylation rates in piglet PBMCs between 79.1 and 82.9%. A high proportion of variation in DNA methylation could be attributed to litter, which emphasized the need to correct for this variable when assessing for weaning-specific differential methylation patterns. The presence of 2,674 DMRs between stages indicates that weaning-associated changes in DNA methylation were present at many genomic loci. Additionally, we observed unique differential methylation patterns between LS and HS animals, with HS animals possessing more DMRs but LS animals having a greater proportion of hypomethylated regions. Previous studies in livestock species have identified differential methylation patterns in stressed versus non-stressed animals. Hao et al. (2016) identified thousands of DMRs in *longissimus dorsi* muscle of heat-stressed versus control pigs within genes involved in energy metabolism, stress response, and calcium signaling. Multiple studies have identified differential lymphocyte DNA methylation associated with prenatal transportation stress in Brahman bulls and heifers (Littlejohn et al., 2018; Baker et al., 2020; Cilkiz et al., 2020) and identified thousands of DM loci enriched in stress and immune response genes. Our results indicate that weaning stress also alters DNA methylation patterns in pig immune cells—particularly within gene promoters—and that the magnitude and direction of such alterations may be dependent on the level of stress experienced.

When considering all animals, post-weaning differential methylation impacted genes involved in immune cell processes. *IL2RA* exhibited hypermethylation in post-weaning PBMCs; this gene has previously been shown to possess extensive promoter hypomethylation in activated CD4+ T cells (Belot et al., 2018), suggesting that the opposite state observed here may suppress such activation. Clear differences were observed when assessing GO enrichment among LS and HS DMGs. Particularly, LS animals exhibited hypomethylation of genes involved in T cell activation and differentiation, while HS animals exhibited hypermethylation of genes involved in these processes. Overall, expression patterns were consistent with the differential methylation observed in LS and HS animals in terms of impacted biological processes. Upregulated HS genes were enriched for GO terms related to T cell apoptosis and negative regulation of CD4+ T cell proliferation, and these were not observed among the LS upregulated genes. Previous studies have shown CD4+ T cell concentrations to be the most significantly reduced following periods of psychosocial and weaning stress (Tuchscherer et al., 2009). Our data suggest that differential gene regulation by DNA methylation may play a role in a reduced T cell response with increasing levels of stress. This was particularly evident when assessing differential methylation and expression by stress group among T cell co-receptor genes. Many of these genes exhibited post-weaning differential methylation patterns indicative of lower gene activation in the HS group compared to the LS group, and *CD8B* also exhibited significantly lower expression post-weaning only in the HS group. *CD8B* encodes a subunit of the CD8 co-receptor in cytotoxic T cells. Studies have shown that corticosterone injections decrease CD8+ T cell concentrations in humans (Nair et al., 2007; Ashcraft et al., 2008) and that in pigs cytotoxic T cell concentrations decrease following weaning (Kick et al., 2012). Our methylation analyses have identified a DMR in the gene body of *CD8B* that may act in regulating *CD8B* expression, particularly in response to weaning stress and cortisol levels.

Lastly, we observed significant post-weaning hypermethylation in CpGs in the *NR3C1* promoter and a corresponding decrease in expression. The glucocorticoid receptor not only functions as an inducer of cortisol-mediated transcription in peripheral tissues but also regulates the HPA axis response in a negative feedback loop (de Kloet et al., 1998). *NR3C1* hypermethylation and downregulation in the hypothalamus has often been an indicator of stress vulnerability, and recent studies have shown comparable patterns in PBMCs (Oberlander et al., 2008; Perroud et al., 2011). Our data show that pigs exhibit similar patterns of *NR3C1* hypermethylation and downregulation in response to weaning stress. However, we did not observe significant differences in post-weaning *NR3C1* methylation and expression between LS and HS pigs, potentially due to our limited sample size in this study.

## Conclusion

In summary, we have elucidated epigenetic patterns of acute weaning-associated stress response in pigs. Future studies should seek to assess patterns of methylation and expression in PBMCs at later periods following weaning to assess long-term effects of weaning stress on pig immunity and performance. Additionally, assessment of other tissues involved in the HPA axis would provide a more direct measurement of stress response that could be compared to regulation and expression of genes in peripheral tissues. Continued analysis of genes undergoing stress-dependent gene regulation and expression may reveal biomarkers that are predictive of pig stress resilience.

## Data Availability Statement

The datasets presented in this study can be found in online repositories. The names of the repository/repositories and accession number(s) can be found below: https://www.ncbi.nlm.nih.gov/, PRJNA632582.

## Ethics Statement

The animal study was reviewed and approved by the Michigan State University Institutional Animal Care and Use Committee AUF# 04/17-062-00.

## Author Contributions

CE and JS designed the experiment. RC, KW, and CE collected the blood samples. KW performed the lesion scoring. RC and NR performed the nucleic acid isolation and immunoassays. RC and AL analyzed the phenotypic and sequencing data. LF performed the RT-qPCR and analyzed the results. RC wrote the manuscript. All authors read, edited, and approved the manuscript.

## Conflict of Interest

The authors declare that the research was conducted in the absence of any commercial or financial relationships that could be construed as a potential conflict of interest.
