# Supplementary figures and images for: Weaning Induces Stress-Dependent DNA Methylation and Transcriptional Changes in Piglet PBMCs
Source: Front Genet. 2021 Feb 5;12:633564. doi: 10.3389/fgene.2021.633564 (PMC7893110; doi:10.3389/fgene.2021.633564)

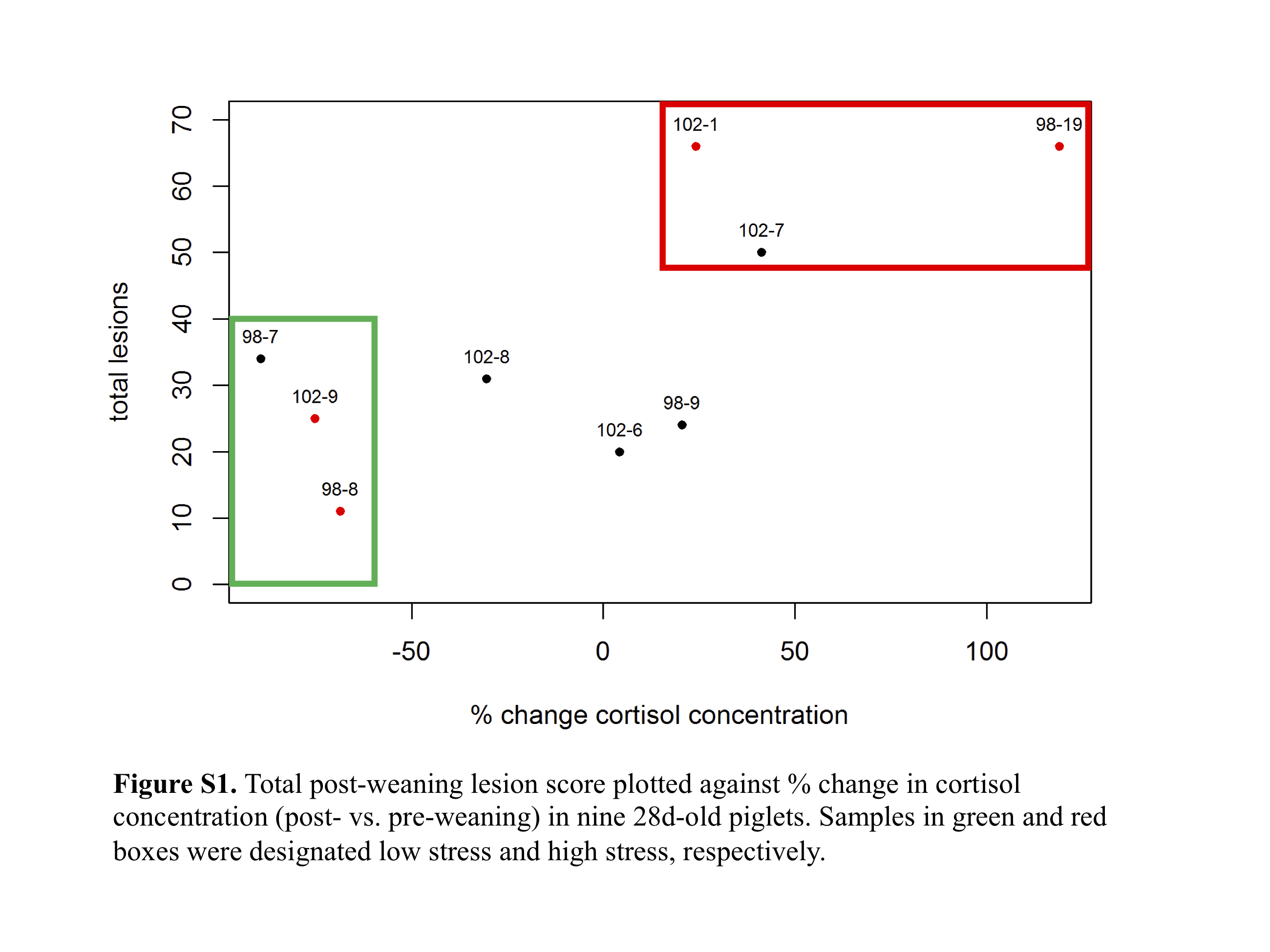

Supplement: Supplementary file 1 [file Image_1.TIFF]

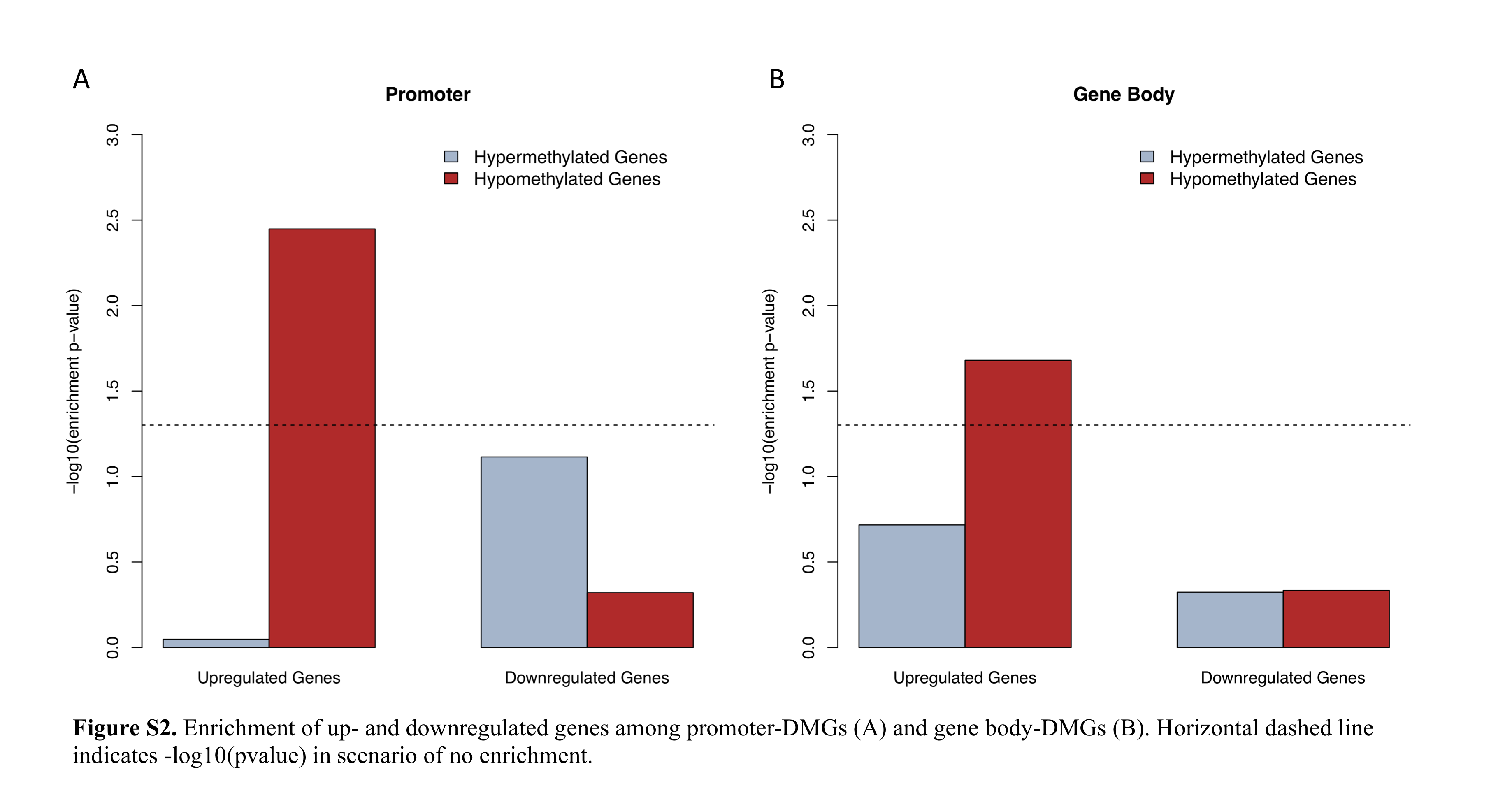

Supplement: Supplementary file 2 [file Image_2.TIFF]
